# The patterns of acceptance, mindfulness, and values for Japanese patients with type 2 diabetes mellitus: a web-based survey

**DOI:** 10.1186/s13030-022-00236-3

**Published:** 2022-03-07

**Authors:** Junichi Saito, Hiroaki Kumano

**Affiliations:** 1 Comprehensive Research Organization, Waseda University, 2-579-15, Mikajima, Tokorozawa, Saitama, 359-1192 Japan; 2 Faculty of Human Science, Waseda University, Tokyo, Japan

**Keywords:** Acceptance and commitment therapy, Mindfulness, Type 2 diabetes

## Abstract

**Background:**

The Acceptance and Commitment Therapy (ACT) model of human functioning uses the behavioral processes of *acceptance*, *mindfulness*, and *values*, which together compose *psychological flexibility*, the ability to contact the present moment more fully as a conscious human being and to either change or persist when doing so serves valued ends. To increase the effectiveness of interventions in the medical treatment of diabetes, it is important to examine the effects on patients with type 2 diabetes of promoting the active component patterns of ACT. This study explores these points.

**Methods:**

Questionnaires were administered to type 2 diabetes patients who were registered in the database of a research service provider, and data was collected and analyzed from a total of 211 patients (mean age ± SD was 58.84 years old ±10.25, 14.69% were females).

**Results:**

Cluster analysis yielded four clusters: “*Average*” (average levels of *acceptance*, *mindfulness*, and *values*), “*Flexibility*” (high levels of *acceptance*, *mindfulness*, and *values*), “*Values/low*” (average levels of *acceptance* and *mindfulness*, and a low level of *values*), “*Values/high*” (average levels of *acceptance* and *mindfulness* and a high level of *values*). Patients in the “*Flexibility*” and “*Values/high*” clusters had significantly fewer depressive symptoms than the other clusters. However, members of the “*Values/high*” cluster demonstrated significantly higher glycated hemoglobin levels than those in the other clusters.

**Conclusions:**

The results above indicate that each part of the ACT model is necessary for managing diabetes treatment while improving quality of life. The importance of *values* is emphasized in ACT for diabetes patients, but we argue, given our results, that *acceptance* and *mindfulness* are very important for Japanese patients with type 2 diabetes. This study is limited to Japanese patients with type 2 diabetes. In further research, the subject population must be expanded to people from other areas and of different racial backgrounds.

## Background

Control of diabetes depends greatly on self-management, meaning that attention must be paid to self-care activities. Factors largely influencing self-care behaviors are the patient’s way of thinking, emotions, stress, diabetes complications, support from medical staff, family, school, work place members, region, and health care systems, etc. Therefore, it is necessary to assess the patient’s psychological and social problems in addition to physical problems to achieve satisfactory glycemic control.

For this reason, many psychological interventions have been conducted for diabetes patients, many of which have proven effective but costly. A meta-analysis found that most such studies used 10 or more treatment sessions and that, on average, 24 h of intervention was needed to reduce HbA1c levels by 1% [1]. Thus, in clinical practice, psychological therapies are generally not used due to the time and effort they require [2].

Recently, Acceptance and Commitment Therapy (ACT) has received increasing recognition as a viable alternative to other psychological therapies [3]. It has been shown that a one-day workshop style of ACT intervention is effective for diseases such as type 2 diabetes, multiple sclerosis [4], migraine [5, 6], obesity [7], vascular disease [8]. These studies suggest that focusing on the ACT behavioral process could possibly solve the problems concerning intervention duration and the amount of effort.

The ACT model of human functioning includes the behavioral processes of *acceptance*, *mindfulness*, and *values*, which together compose *psychological flexibility*, the ability to contact the present moment more fully as a conscious human being and to either change or persist when doing so serves valued ends [9]. *Acceptance* is the voluntary adoption of an intentionally open, receptive, flexible, and nonjudgmental posture with respect to moment-to-moment experience. *Mindfulness* helps one consciously center oneself in the here and now. It is a grounded awareness of one’s experience instead of identifying with it, resisting it, or rejecting it. *Values* promotes awareness of positive reinforcement that one is engaging in acting according to one’s values and enables one to focus on processes rather than results, motivating one to pursue personal goals.

Most psychological studies involving the application of cognitive-behavior therapy have concentrated on decreasing, changing, or stopping negative thoughts and emotions related to diabetes. However, the ACT model focuses on the acceptance of negative thoughts and emotions, which emphasizes values and personal goals [9].

One study found that patients receiving a one-day ACT intervention were more likely to report superior self-care activities and have glycated hemoglobin (HbA1c) values in the target range of under 7% 3 months after the intervention than patients who only received diabetes education [10]. Mediational analysis showed that increases in *acceptance* mediated improvement in HbA1c values. Because of the intervention effects of a one-day ACT workshop, as indicated by that study, the introduction of psychological therapeutic interventions into the medical treatment of diabetes on reasonable terms can be expected to become a direction of therapy. A one-day workshop ensures treatment adherence and completion, the lack of which is often the greatest obstacle to effective delivery of mental health services. In fact, a meta-analysis of 125 studies of outpatient psychotherapy found that 50% of patients prematurely terminate participation, with nearly 40% dropping out after only the first or second visit [11, 12].

*Psychological flexibility* includes the behavioral processes of *acceptance*, *mindfulness*, and *values*; however, the above-mentioned study assessed *acceptance* but not *mindfulness*, and *values*. It is not yet known how these behavioral processes relate to one another in support of their mutual effectiveness. To create more efficient interventions that are suited to the medical treatment of diabetes, it is important to examine the retention pattern of behavioral processes of ACT relative to diabetes. An emphasis on *values* may be more relevant for medical conditions such as diabetes that require significant self-management [12]. This study explores these points.

## Methods

### Participants

To gather data from a wide range of community samples, we used an online survey, conducted with the assistance of a marketing research service provider. From among approximately 13,203 responses by Japanese patients with type 2 diabetes registered in the database of the research service provider, we obtained valid responses from 211 individuals (mean age 58.84 years ±10.25, 14.69% female, mean illness duration 10.62 years ±8.41, rate of complication 18.14, 24.18% had cessation of treatment).

### Measures

#### Demographics

Sociodemographic information on age, sex, complications, and treatment status were obtained via the self-reported questionnaires.

#### Acceptance

The Acceptance and Action Diabetes Questionnaire (AADQ), an 11-item instrument, measures the acceptance of diabetes-related thoughts and feelings and the degree to which they interfere with valued action [9]. The Japanese version of AADQ was used [13]. For psychometric concerns, three items were excluded, leaving eight-items for analysis. Respondents rated the items on a 7-point Likert scale (1 = never true of me to 7 = always true of me).

#### Mindfulness

The Mindful Attention Awareness Scale (MAAS) is a 15-item instrument that measures to what extent respondents pay mindful attention to and are aware of their present experiences [14]. If we do not pay deliberate attention to our thoughts and feelings, which is a function of the observer self, we will almost automatically act. For example, if you notice a high-calorie food in front of you, you will eat it, or if the TV is on, you will sit down and watch without thinking. The MAAS includes items such as “I snack without being aware that I’m eating.” It is an appropriate scale to measure the mindfulness of diabetes patients. In this study, the Japanese version of MAAS was used [15]. The respondents rated the items on a 7-point Likert scale (0 = almost always to 6 = almost never).

#### Values

The Values Clarification Questionnaire for Patients with Diabetes (VCQD) is a 15-item instrument that measures progress toward living one’s values [16, 17]. The questionnaire has excellent internal consistency (*α* = .90) and has criterion-related validity with the Valuing Questionnaires [18, 19] and the Short Form-8 Health Questionnaire (SF-8) [20, 21]. The statistical results were as follows (Valuing Questionnaires Progress: *r* = .58, Valuing Questionnaires Obstruction: *r* = −.22, SF-8 physical-related quality of life: *r* = .30, SF-8 mental-related quality of life: *r* = .27). In this study, the respondents composed a diabetes-related value description following the instruction given and then rated items on a 7-point Likert scale (0 = not at all true of me to 6 = completely true of me) to answer the questions. The instructions, as well as examples of items, are given in the following. Instructions: Write down why and for what purpose/reason you would alter your lifestyle. Refer to the following questions. What kind of life would you like to live if your disease stopped progressing and your body were as free as you wish? What way of spending your time and energy would make you happiest? Examples: “It is rewarding to take steps in accordance with my values even though I have difficulties” and “I feel vigorous when taking steps in accordance with my values.”

#### Diabetes self-management

The Summary of Diabetes Self-Care Activities Measure (SDSCA) is a 17-item instrument that measures the frequency of the performance of diabetes self-care over the last 7 days, including diet, exercise, blood glucose testing, foot care, and tobacco use [22]. In this study, the subscales of the Japanese version of SDSCA on diet and exercise were used [23]. The respondents marked the number of days on which the indicated behavior was performed on an 8-point Likert scale (from 0 to 7 days).

#### Diabetes-specific distress

The Problem Areas in Diabetes Treatment Satisfaction Questionnaire (PAID) is a 20-item instrument that measures diabetes-specific distress [24]. In this study, the Japanese version of PAID was used [25]. The respondents rated items on a 5-point Likert scale (0 = not a problem for me to 4 = a serious problem for me).

#### Depressive symptoms

The Center for Epidemiologic Studies Depression Scale (CES-D) is a 20-item instrument that measures depressive symptoms in the general population [26]. In this study, the Japanese version of CES-D was used [27]. The respondents rated items on a 4-point Likert scale (0 = rarely or none of the time to 3 = most or all of the time).

#### Hemoglobin A1c

Patients’ hemoglobin A1c (HbA1c) levels were given as self-reported data. HbA1c is the most common assessment of glycemic control. It reflects average blood glucose over the past 3 months.

### Statistical analysis

Single regression analysis, model-based cluster analysis, and one-way analyses of covariance (ANCOVA) were conducted in three steps. All analyses were conducted using HAD16_202 [28].

Step 1: Single regression analyses were conducted to examine age, sex, duration of illness, complications, and cessation of treatment as possible predictors of diabetes-related outcomes.

Step 2: Improved *k*-means cluster analysis was conducted to derive the patterns for *acceptance*, *mindfulness*, and *values* for Japanese patients with type 2 diabetes. Improved *k*-means cluster analysis was originally developed as an improvement to traditional cluster analyses that allows test and comparisons of the fit of a number of cluster solutions using log likelihood, the Bayesian Information Criteria (BIC) and the Akaike Information Criteria (AIC) as indicators of the relative fit of different cluster solutions. Substantive differences in patterns within the clusters were also considered.

Step 3: After determining the optimal number of clusters, one-way ANCOVA controlling for age, sex, duration of illness, complications, and cessation of treatment found in the preliminary analyses, were conducted to test for differences in diabetes-related outcomes among clusters. Post-hoc tests were conducted using the Holm method, used to control for type I errors. In addition, the index for cohen’s *d* was calculated in effect size, serving as a standardized indicator unaffected by sample size.

### Ethical considerations

This study was approved by the Waseda University Academic Research Ethical Review Committee (2017–227). Informed consent was obtained by all the study participants. The study protocol followed the guidelines for Epidemiological Studies in accordance with the Declaration of Helsinki. The authors declare that they have no conflict of interest.

## Results

In the first step, preliminary single regression analyses were conducted to determine which demographic characteristics were predictive of outcomes. These included the direct effects of age, sex, duration of illness, complications, and cessation of treatment (Table 1). Age predicted SDSCA diet (*β* = 0.21; *p* < .01), SDSCA exercise (*β* = 0.25; *p* < .01), PAID (*β* = − 0.19; *p* < .01), and CES-D (*β* = − 0.39; *p* < .01). Sex (female) predicted SDSCA diet (*β* = 0.18; *p* < .01). Cessation of treatment (yes) predicted CES-D (*β* = − 0.19; *p* < .01). Complications (yes) predicted PAID (*β* = 0.30; *p* < .01).

**Table 1:** Results of the simple linear regression model

Variables	*β*	95% CI	*P* value
Diet			
Age	.21	[.08, .35]	.00
Sex	.18	[.05, .32]	.01
Illness duration	.02	[−.12, .32]	.83
Complication	.02	[−.12, .16]	.78
Cessation of treatment	.06	[−.08, .19]	.43
Exercise			
Age	.25	[.11, .38]	.00
Sex	−.03	[−.17, .11]	.63
Illness duration	.01	[−.13, .15]	.86
Complication	−.11	[−.25, .03]	.13
Cessation of treatment	.04	[−.01, .18]	.55
PAID			
Age	−.19	[−.32, −.05]	.00
Sex	−.09	[−.23, .05]	.19
Illness duration	−.01	[−.15, .13]	.91
Complication	.30	[.17, .43]	.00
Cessation of treatment	.01	[−.13, .15]	.86
CES-D			
Age	−.39	[−.51, −.26]	.00
Sex	.00	[−.13, .15]	.92
Illness duration	−.11	[−.25, .03]	.11
Complication	.13	[−.01, .26]	.07
Cessation of treatment	−.19	[−.33, −.05]	.01
HbA1c			
Age	.12	[−.01, .26]	.08
Sex	−.14	[−.27, .00]	.06
Illness duration	.12	[−.02, .26]	.09
Complication	.09	[−.05, .23]	.21
Cessation of treatment	.13	[−.01, .27]	.06

*Note*. *β* = Standardized regression coefficients, Diet = The Summary of Diabetes Self-Care Activity Measure subscale of diet, Exercise = The Summary of Diabetes Self-Care Activity Measure subscale of exercise, PAID = The Problem Areas in Diabetes Treatment Satisfaction, CES-D = The Center for Epidemiologic Studies Depression. Sex is coded (0 = Male; 1 = Female), Complication is coded (0 = No; 1 = Yes), Cessation of treatment is coded (0 = No; 1 = Yes)

**Fig. 1:**
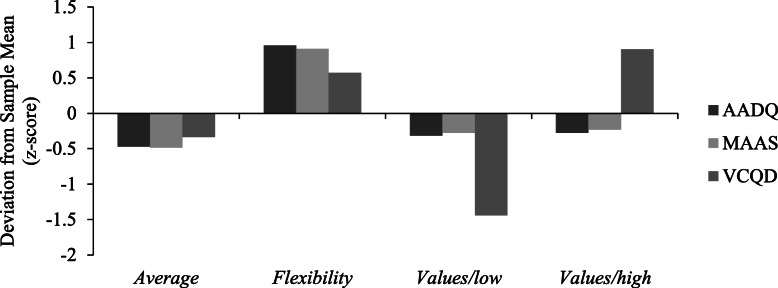
Patterns of Acceptance, Mindfulness and Values by Differences from the Sample Grand Mean (Z-Score). *Note*. AADQ = The Acceptance and Action Diabetes Questionnaire, MAAS = The Mindful Attention Awareness Scale, VCQD = The Values Clarification Questionnaire for Patients with Diabetes

**Fig. 2:**
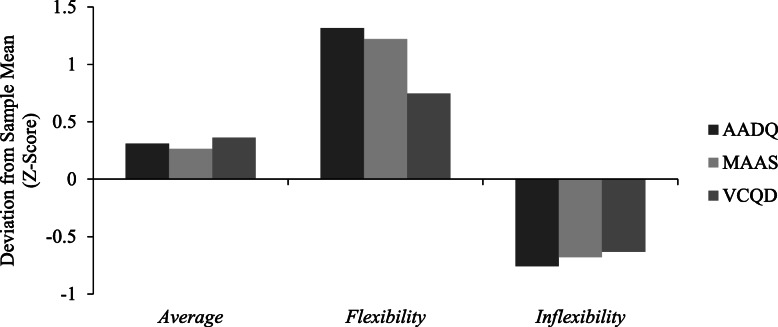
Patterns of Acceptance, Mindfulness and Values by Differences from the Sample Grand Mean (Z-Score). *Note*. AADQ = The Acceptance and Action Diabetes Questionnaire, MAAS = The Mindful Attention Awareness Scale, VCQD = The Values Clarification Questionnaire for Patients with Diabetes

In the second step, improved *k*-means cluster analysis was conducted to derive the retention patterns for *acceptance* (AADQ), *mindfulness* (MAAS), and *values* (VCQD) for Japanese patients with type 2 diabetes. Three- to six-class models with equal indicator variances were estimated without problems. Using solely fit statistics (log likelihood, BIC, and AIC), as the number of clusters increased and the retention pattern of behavioral processes became more subdivided, the model fit became worse. The three-class model was found to be the statistically preferred model; however, it is also important to consider substantive differences in patterns within clusters. The four-class model provided four characteristic patterns with clearly differentiated *values* between the clusters (Fig. 1). In contrast, because z-scores of three scales in the three-class model were similar in each cluster, the clusters were merely average, high, and low in all scales without significant information of behavioral processes related to ACT (Fig. 2). In the identification of possible intervention targets, it was considered optimal to determine four distinctly different process patterns. Taking into consideration both fit statistics and process patterns, the freed-variance four-class model was found to be the most appropriate (Table 2). Standardized z-scores were calculated for each measure within each cluster to examine the conceptual differences among clusters. Labels for the clusters were created by examining these differences. Cluster 1 (“*Average*” *N* = 66) described patients who had average levels (− 0.50 < z-score < 0.50) of *acceptance*, *mindfulness*, and *values*. Cluster 2 (“*Flexibility*” *N* = 59) described patients who exhibited high levels (z-score > 0.50) of *acceptance*, *mindfulness*, and *values*. Cluster 3 (“*Values/low*” *N* = 38) described patients who exhibited average levels (− 0.50 < z-score < 0.50) of *acceptance* and *mindfulness* and low levels (z-score < − 0.50) of *values*. Cluster 4 (“*Values/high*” *N* = 48) described patients who exhibited average levels (− 0.50 < z-score < 0.50) of *acceptance*, *mindfulness,* and high levels (z-score > 0.50) of *values*. In this study, a z-score higher than 0.50 was defined as “*high*” and a score lower than − 0.50 as “*low*”. The mean scores of the AADQ, MAAS, and VCQD in each cluster were as follows. In cluster 1, AADQ 39.20, MAAS 62.33, VCQD 61.55; in cluster 2, AADQ 48.78, MAAS 77.70, VCQ 77.25; in cluster 3, AADQ 40.24, MAAS 64.63, VCQD 42.45; and in cluster 4 AADQ 40.50, MAAS 65.15 and VCQD 83.55.

**Table 2:** Fit Statistics of Clusters Derived via Improved *k*-means Cluster Analysis

Number of Clusters	Log likelihood	AIC	BIC	SBIC
3	− 2383.44	4806.89	4873.92	4810.55
4	− 2379.04	4812.08	4902.58	4817.03
5	− 2372.80	4813.61	4927.57	4819.84
6	− 2373.96	4829.92	4967.35	4837.44

*Note*. *AIC* Akaike’s Information Criterion, *BIC* Bayesian information criterion, *SBIC* Schwartz Bayesian information criterion

The third step, a one-way ANCOVA controlling for significant interactions found in the preliminary analyses was conducted to test for cluster differences (Table 3). The ANCOVA for SDSCA diet and exercise did not produce significant results: *F* (3, 199) = 0.71, *p* = *n.s*.; *F*(3, 203) = 1.46, *p* = *n.s*. The ANCOVA for diabetes-specific distress (PAID) showed a significant trend, *F*(3, 195) = 2.24, *p* < .10. Using a Bonferroni correction, the results of post-hoc tests revealed that “*Average”* demonstrated more diabetes-specific distress than “*Flexibility”* (*p* < .01, *d* = 0.90), that “*Values/low”* demonstrated more diabetes-specific distress than “*Flexibility*,*”* (*p* < .01, *d* = 0.85) and that “*Values/high”* demonstrated more diabetes-specific distress than “*flexibility*” (*p* < .01, *d* = 0.68). The ANCOVA for depressive symptoms (CES-D) was significant: *F*(3, 203) = 3.07, *p* < .03. Using a Bonferroni correction, the results of post-hoc tests revealed that “*Values/low*” demonstrated more depressive symptoms than “*Average*” (*p* < .00, *d* = 0.67), “*Average*” demonstrated more depressive symptoms than “*Flexibility*” (*p* < .00, *d* = 1.18), “*Values/low*” demonstrated more depressive symptoms than “*Flexibility*” (*p* < .00, *d* = 1.85), and “*Values/high*” demonstrated more depressive symptoms than “*Flexibility*” (*p* < .01, *d* = 0.59). “*Average*” demonstrated more depressive symptoms than “*Values/high*” (*p* < .01, *d* = 0.59), and “*Values/low*” demonstrated more depressive symptoms than “*Values/high*” (*p* = .00, *d* = 1.26). The ANCOVA for HbA1c was significant: *F*(3, 203) = 2.64, *p* < .05. Using a Bonferroni correction, the results of post-hoc tests showed that “*Values/high*” demonstrated much higher HbA1c than “*Flexibility”* (*p* < .00, *d* = 0.77).

**Table 3:** Means, Standard Error, and Holm /Post-hoc tests ANCOVA Results

	Mean values (Standard Error)				ANCOVA	Holm /Post-hoc tests ANCOVA	
	Cluster 1 *Average* (N = 66)	Cluster 2 *Flexibility* (N = 59)	Cluster 3 *Values/low* (N = 38)	Cluster 4 *Values/high* (N = 48)	*p* value	Pairwise comparisons	Cohen’*d*
Diet	21.20 (0.71)	26.14 (0.78)	19.32 (1.01)	24.18 (0.87)	0.55	*–*	*–*
Exercise	7.39 (0.48)	8.37 (0.51)	5.84 (0.68)	8.30 (0.58)	0.23	*–*	*–*
PAID	47.28 (1.61)	35.56 (1.73)	46.56 (2.28)	44.32 (1.95)	0.09	*Flexibility* < *Average*	0.90
						*Flexibility* < *Values/low*	0.85
						*Flexibility* < *Values/high*	0.68
CES-D	17.73 (0.92)	8.86 (0.98)	22.82 (1.22)	13.29 (1.08)	0.01	*Average < Values /low*	0.67
						*Flexibility* < *Average*	1.18
						*Flexibility < Values/low*	1.85
						*Flexibility* < *Values/high*	0.59
						*Values/high < Average*	0.59
						*Values/high < Values/low*	1.26
HbA1c	6.84 (0.10)	6.50 (0.10)	6.64 (0.14)	7.10 (0.12)	0.05	*Flexibility* < *Values/high*	0.77

*Note*. Diet = The Summary of Diabetes Self-Care Activity Measure subscale of diet, Exercise = The Summary of Diabetes Self-Care Activity Measure subscale of exercise, PAID = The Problem Areas in Diabetes Treatment Satisfaction, CES-D = The Center for Epidemiologic Studies Depression

## Discussion

This study investigated empirically derived patterns for *acceptance*, *mindfulness*, and *values* in a sample of Japanese patients with type 2 diabetes as indicators of *psychological flexibility* to examine the relations among these patterns and diabetes-related outcomes.

Regarding the influence demographic data have on diabetes related outcomes, single regression analysis results indicated that age is related to many diabetes related outcomes. The higher the age is, self-care behaviors such as dietary management and exercise are apt to be continued and depressive symptoms and diabetes related distress to be lower. It is reported that patients in late middle age are troubled by not being able to prioritize their dietary management because of “business relationships”, which includes eating and drinking with business partners [29]. It might be that this studies’ participants tended to experience difficulty in maintaining self-care behaviors, depressive symptoms, and diabetes related distress in their everyday lives because their average age was 58.84; middle-aged patients in their forties to fifties are busy with their work and engage in frequent wining and dining compared with older patients, including retirees in their sixties to seventies.

Also, it is indicated that the onset of diabetes complications is related to diabetes related distress, with patients having diabetes complications expressing stronger diabetes related distress compared with patients with no complications. A survey using PAID (diabetes related distress) given to 653 diabetes outpatients demonstrated that age, female sex, medication (especially insulin treatment), complications, hospitalization, hypoglycemia, and high HbA1c predicted higher PAID scores [30]. Some of these variables were not measured in this study, but similar results were found in terms of lower diabetes-related distress with older age and higher diabetes-related distress in patients with diabetic complications. Improved *k*-means cluster analysis results identified four clusters, Cluster 1 “*Average*” (average levels of *acceptance*, *mindfulness*, *and values*), Cluster 2 “*Flexibility*” (high levels of *acceptance*, *mindfulness*, and *values*), Cluster 3 “*Values/low*” (average levels of *acceptance* and *mindfulness* and a low level of *values*), and Cluster 4 “*Values/high*” (average levels of *acceptance* and *mindfulness* and a high level of *values*). *Acceptance *and *mindfulness* were at similar levels (z < .05) in Cluster 1 “*Average*”, Cluster 3 “*Values/low*”, and Cluster 4 “*Values/high*”. In other words, these clusters were classified according to the level of *values*. These results indicate that while a number of patients with only high levels in clarified values exist, there are only few patients showing high levels only in acceptance and mindfulness. In addition, it was also proven that while acceptance and mindfulness had a strong relation, this behavioral process had a weak relation with values clarification. This indicates that the ACT behavioral process is largely divided into the “mindfulness and acceptance process” and the “commitment and behavioral changing process”.

ANCOVA results showed that “*Flexibility*” had lower diabetes-specific distress and depressive symptoms compared with the other clusters. Thus, increasing flexibility via ACT interventions can improve quality of life among type 2 diabetes patients. However, no main effects of clusters were shown for self-care behaviors, such as diet and exercise. The difficulty in measuring self-care behaviors such as diet and exercise via retrospective questionnaires might be related to this result. Prince et al. (2008) conducted a systematic review of the measurement of physical activity and found that retrospective questionnaires and direct measures (e.g., accelerometers) were not consistent [31]. Measurements using retrospective questionnaires of everyday behaviors such as diet and exercise are susceptible to recall bias and therefore include the probability of undermined ecological validity. SDSCA is a globally used instrument to measure self-care behaviors, but the low relation its results have with glucose control levels has been noted [32, 33]. In diet, the mean for “*Flexibility*” with the most favorable HbA1c was 26.14, and the mean for “*Value/high*” with the most unfavorable HbA1c was 24.18, with little difference. Similarly, there was little difference in exercise, with the mean for “*Flexibility*” being 8.38 and the mean for “*Value/high*” 8.30. Therefore, little association was shown between diet, exercise in SDSCA and HbA1c (*r* = −.11, *r* = − 04). Based on these results, it may be necessary to measure self-care behavior using methods such as Ecological Momentary Assessment that record in real time [34]. For example, calorie intake can be calculated from a food diary using a Personal Digital Assistant (PDA), and exercise intensity (METs; Metabolic Equivalent of Task) can be calculated from physical activity using an accelerometer. These may show a better relation with HbA1c than retrospective questionnaires. Furthermore, the EMA will allow us to examine the true effectiveness of the ACT process. For example, we must examine whether patients with high levels of *psychological flexibility* tend to accept diabetes-related unpleasant thoughts and emotions occurring before meals, thus leading them to continue their appropriate caloric intake. We must also examine whether patients with high levels of *value* tend to exercise appropriately according to their own values while having negative thoughts and feelings such as “I am too lazy to move after eating”.

“*Average*” showed the same level of diabetes-related distress as “*Values/low*” and “*Values/high*”. The mean CES-D score for “*Average*” was 17.73 points; the cutoff for CES-D was 16 points and “*Average*” was above the cutoff [26]. The reason for this is that 32.4% of diabetic patients have depressive comorbidity, and depression is common among diabetic patients [35]. In Japan, Shumiya et al. measured CES-D in 75 patients who participated in a diabetes class [36]. The results showed that the mean was 15.8 (16.9 for men and 15.1 for women), which is around the cutoff and comparable to the “Average” of this study.

“*Values/low*” demonstrated greater degrees of depressive symptoms compared with other clusters. The average score of CES-D for Cluster 3 “*Values*/*Low*” was 23, far exceeding the cutoff. “*Values/high*” demonstrated fewer depressive symptoms than “*Values/low*”. However, diabetes-related distress was similar between “*Values/high*” and “*Values/low*” and HbA1c were the worst for “*Values/high*” among all clusters. This suggests that patients with a pattern of only high levels of *value* and not high levels of *acceptance* and *mindfulness* were not able to face the treatment of diabetes. They live a relatively full life, but their various activities may include many behaviors that worsen their glycemic control. On the other hand, patients with a pattern of low levels of *value* and not high levels of *acceptance* and *mindfulness* may have lower overall activity levels, including behaviors that may worsen glycemic control. This may have resulted in strong depressive symptoms, but not so poor HbA1c.

The importance of *values* is emphasized in ACT for diabetes patients, but we argue, given our results, that *acceptance* and *mindfulness* are very important, at least for Japanese patients with type 2 diabetes.

This study indicated that each aspect of the ACT model is necessary for managing diabetes treatment while improving quality of life. However, considering that patients who demonstrate a high level of *values* only exist in a certain number while patients who demonstrate high levels of only *acceptance* and *mindfulness* do not exist, it might be helpful to emphasize *acceptance* and *mindfulness* in one-day ACT workshops.

The limitations of this study are as follows. Firstly, because it was carried out by internet survey, items such as sociodemographic information and HbA1c were based on the participant’s self-report. This may be the reason for the low rate of diabetes complications. In addition, many mildly ill patients with relatively good HbA1c were included. Therefore, the results may not be generalizable to critically ill patients with poorer HbA1c. Secondly, the subjects of this study were limited to Japanese patients with type 2 diabetes. Type 2 diabetes is a disease showing wide disparities due to race and environment. For example, the average Japanese type 2 diabetes patient is less obese than the average in Europe and the United States. In further studies, we must extend our research subjects to include people from different areas and of different races. Thirdly, there was limited information on the attributes of the subjects. For example, we considered that employment status was related to the fact that older people were more likely to continue self-care behaviors such as diet control and exercise. However, we did not measure variables related to employment status, which limits our ability to examine this point. Self-care behavior is thought to be related to various attributes of the subjects. In the future, it will be necessary to examine the relation between psychological flexibility and self-care behavior by considering a wider range of subject attributes in addition to age, sex, duration of illness, complications, and treatment status.

## Conclusions

The purpose of the present study was to examine the effects of promoting the active component patterns of ACT on Japanese patients with type 2 diabetes. The findings of the present study indicate that patients who have “*Flexibility*” and “*Values/high*” had lower depressive symptoms. However, “*Values/high*” demonstrated diabetes-related distress and poor blood-glucose levels. *Values* is emphasized in ACT for diabetes patients, but this study indicated that *acceptance* and *mindfulness* are very important for Japanese patients with type 2 diabetes. It might be helpful to emphasize *acceptance* and *mindfulness* in one-day ACT workshops.

## Data Availability

The datasets generated and analyzed are not publicly available due to the regulations of the ethics committee.

## Abbreviations

AADQ: The Acceptance and Action Diabetes Questionnaire
ACT: Acceptance and Commitment Therapy
AIC: Akaike Information Criteria
ANCOVA: Analyses of Covariance
BIC: Bayesian Information Criteria
CES-D: The Center for Epidemiologic Studies Depression Scale
MAAS: The Mindful Attention Awareness Scale
PAID: The Problem Areas in Diabetes Treatment Satisfaction Questionnaire
SDSCA: The Summary of Diabetes Self-Care Activities Measure
SF-8: The Short Form-8 Health Questionnaire
VCQD: The Values Clarification Questionnaire for Patients with Diabetes
